# Association between HIV stigma and antiretroviral therapy adherence among adults living with HIV: baseline findings from the HPTN 071 (PopART) trial in Zambia and South Africa

**DOI:** 10.1111/tmi.13473

**Published:** 2020-08-26

**Authors:** Harriet S. Jones, Sian Floyd, Anne Stangl, Virginia Bond, Graeme Hoddinott, Triantafyllos Pliakas, Justin Bwalya, Nomtha Mandla, Ayana Moore, Deborah Donnell, Peter Bock, Sarah Fidler, Richard Hayes, Helen Ayles, James R. Hargreaves, the HPTN 071 (PopART) Study Team

**Affiliations:** ^1^ Department of Public Health, Environments and Society London School of Hygiene and Tropical Medicine London UK; ^2^ Department of Infectious Disease Epidemiology London School of Hygiene and Tropical Medicine London UK; ^3^ International Center for Research on Women Washington DC USA; ^4^ Zambart School of Medicine University of Zambia Lusaka Zambia; ^5^ Department of Global Health and Development London School of Hygiene and Tropical Medicine London UK; ^6^ Desmond Tutu TB Centre Stellenbosch University Tygerberg South Africa; ^7^ FHI 360 Durham NC USA; ^8^ Statistical Center for HIV/AIDS Research and Prevention Fred Hutchinson Cancer Research Center Seattle WA USA; ^9^ Department of Medicine Imperial College London London UK; ^10^ Department of Clinical Research London School of Hygiene and Tropical Medicine London UK

**Keywords:** human immunodeficiency virus, antiretroviral therapy, treatment adherence, stigma, South Africa, Zambia, virus de l'immunodéficience humaine, thérapie antirétrovirale, adhésion au traitement, stigmatisation, Afrique du Sud, Zambie

## Abstract

**Objectives:**

Adherence to antiretroviral therapy (ART) leads to viral suppression for people living with HIV (PLHIV) and is critical for both individual health and reducing onward HIV transmission. HIV stigma is a risk factor that can undermine adherence. We explored the association between HIV stigma and self‐reported ART adherence among PLHIV in 21 communities in the HPTN 071 (PopART) trial in Zambia and the Western Cape of South Africa.

**Methods:**

We conducted a cross‐sectional analysis of baseline data collected between 2013 and 2015, before the roll‐out of trial interventions. Questionnaires were conducted, and consenting participants provided a blood sample for HIV testing. Poor adherence was defined as self‐report of not currently taking ART, missing pills over the previous 7 days or stopping treatment in the previous 12 months. Stigma was categorised into three domains: community, health setting and internalised stigma. Multivariable logistic regression was used for analysis.

**Results:**

Among 2020 PLHIV self‐reporting ever taking ART, 1888 (93%) were included in multivariable analysis. Poor ART adherence was reported by 15.8% (*n* = 320) of participants, and 25.7% (*n* = 519) reported experiencing community stigma, 21.5% (*n* = 434) internalised stigma, and 5.7% (*n* = 152) health setting stigma. PLHIV who self‐reported previous experiences of community and internalised stigma more commonly reported poor ART adherence than those who did not (aOR 1.63, 95% CI 1.21 −2.19, *P* = 0.001 and aOR 1.31, 95% CI 0.96–1.79, *P* = 0.09).

**Conclusions:**

HIV stigma was associated with poor ART adherence. Roll‐out of universal treatment will see an increasingly high proportion of PLHIV initiated on ART. Addressing HIV stigma could make an important contribution to supporting lifelong ART adherence.

## Abstract (fr)

**Objectifs:**

L'adhésion à la thérapie antirétrovirale (ART) conduit à la suppression virale pour les personnes vivant avec le VIH (PVVIH) et est essentielle à la fois pour la santé individuelle et pour réduire la transmission du VIH. La stigmatisation du VIH est un facteur de risque qui peut compromettre l’adhésion. Nous avons exploré l'association entre la stigmatisation du VIH et l'adhésion autodéclarée à l’ART chez les PVVIH dans 21 communautés dans l'essai HPTN 071 (PopART) en Zambie et dans le Western Cape en Afrique du Sud.

**Méthodes:**

Nous avons effectué une analyse transversale des données de base collectées entre 2013‐2015, avant le déploiement des interventions d'essai. Des questionnaires ont été réalisés et les participants consentants ont fourni un échantillon de sang pour le dépistage du VIH. Une mauvaise adhésion a été définie comme l'autodéclaration de ne pas prendre actuellement l’ART, d'omettre des comprimés au cours des 7 jours précédents ou d'arrêter le traitement au cours des 12 mois précédents. La stigmatisation a été classée en trois domaines: communautaire, en milieu de santé et stigmatisation intériorisée. Une régression logistique multivariée a été utilisée pour l'analyse.

**Résultats:**

Parmi les 2.020 PVVIH autodéclarant avoir déjà pris un ART, 1.888 (93%) ont été inclus dans l'analyse multivariée. Une mauvaise adhésion à l’ART a été signalée par 15,8% (*n* = 320) des participants, 25,7% (*n* = 519) ont déclaré avoir subi une stigmatisation communautaire, 21,5% (*n* = 434) une stigmatisation internalisée et 5,7% (*n* = 152) une stigmatisation en milieu de santé. Les PVVIH qui ont auto‐déclaré des expériences antérieures de stigmatisation communautaire et intériorisée ont plus souvent rapporté une mauvaise adhésion à l’ART que ceux qui ne l'ont pas fait (aOR 1,63 ; IC95%: 1,21–2,19 ; *P* = 0,001 et aOR 1,31 ; IC95%: 0,96–1,79 ; *P* = 0,09).

**Conclusions:**

La stigmatisation du VIH était associée à une mauvaise adhésion à l’ART. Le déploiement du traitement universel verra une proportion de plus en plus élevée de PVVIH initiées à l’ART. Lutter contre la stigmatisation du VIH pourrait apporter une contribution importante au soutien de l'adhésion à l’ART au cours de la vie.

**Numéro d'essai clinique:**

NCT01900977

## Introduction

For people living with HIV (PLHIV), adherence to antiretroviral therapy (ART) is crucial for viral suppression [1, 2, 3] and reducing HIV‐related morbidity and mortality [4], onward transmission [5, 6, 7] and drug resistance [8]. UNAIDS 90‐90‐90 targets captured the importance of achieving high levels of HIV testing and ART coverage, with the ‘third 90’ target being that by 2020 90% of those on ART were virally suppressed [9]. In 2016, an estimated 89% of PLHIV in Zambia who reported current ART use [10] and 85% of those registered in HIV care and taking ART in South Africa [11] were virally suppressed. Understanding the factors that influence adherence to ART is crucial if high levels of viral suppression are to be sustained and increased.

HIV stigma can undermine ART adherence [12, 13, 14, 15, 16, 17] and is a frequently reported barrier to adherence in sub‐Saharan Africa [13]. HIV stigma is common in both Zambia and South Africa, with over 35% of PLHIV reporting some type of stigma [18]. Whilst ART adherence is consistently found to be worse among individuals experiencing stigma than among those who do not [19, 20, 21, 22, 23, 24, 25], a 2013 review concluded that all but one study was at risk of bias, and most had not used validated exposure or outcome measures [19]. Currently, data come mostly from facility‐based or purposively sampled populations, and there is heterogeneity in the measurement of both ART adherence and HIV stigma.

We analysed baseline data from the HPTN 071 (PopART) trial [26, 27] to explore the association between HIV stigma and ART adherence for adults with HIV in a random population sample from 21 urban and peri‐urban communities in Zambia and the Western Cape of South Africa. Data were collected between 2013 and 2015, after more than 10 years of scale‐up of HIV treatment services and ART in both countries. We explored these associations among individuals who started ART prior to the implementation of the PopART universal test and treat (UTT) interventions.

## Methods

HPTN071 (PopART) was a cluster‐randomised trial conducted in Zambia and South Africa to assess the impact of a combination of HIV prevention interventions, including household‐based HIV testing and an offer of universal ART initiation regardless of CD4 count or clinical stage for those testing HIV‐positive, on HIV infection rates. Twenty‐one urban communities were purposively selected for inclusion in the trial if they had a heath facility offering HIV and TB services, high HIV prevalence and a population of >20 000. In each country, study communities were matched in triplets based on HIV prevalence and geographic proximity and then randomised to one of three trial arms [26, 27].

Between November 2013 and March 2015, approximately 2000 individuals were enrolled in each study community as a *‘population cohort’* to assess the effect of trial interventions on primary and secondary outcomes. From a simple random sample of households, household members were enumerated and one adult (18–44 years) per household randomly selected for inclusion in the cohort. Selected adults were asked for consent to enrol in the study and participate in a baseline survey and three follow‐up surveys. For those giving consent, a venous blood sample was taken and analysed in‐country using a single fourth‐generation serologic assay. A second fourth‐generation assay was used to confirm HIV‐positive results, and any discrepancies tested with additional assays to confirm HIV status. The baseline survey was conducted using face‐to‐face interviewer administered questionnaires, with data collected on electronic devices. Participants were asked about their HIV status and, if they were happy to do so, share the results of their last HIV test. All participants were offered an on‐the‐spot rapid HIV test.

Our analysis was restricted to individuals who self‐reported living with HIV, with confirmation from the laboratory HIV testing. Among this group, individuals were included if they reported ever starting ART before the 1 January 2014. We excluded participants if they had no information on the year of starting ART or reported starting ART for the prevention of mother to child transmission of HIV (PMTCT) but were no longer taking it, as this may have been due to earlier initiation guidelines and not reflect non‐adherence. We excluded respondents if they had incomplete outcome data or missing data on all stigma questions.

We created a primary outcome variable from three survey questions on ART adherence. We defined poor adherence as ‘respondents self‐reporting that they had ever started ART but were not currently taking ART, or currently taking ART but had either stopped in the past 12 months, or missed pills in the past seven days’. To explore whether our findings were sensitive to our primary definition of adherence, we looked at a secondary outcome, restricting our definition to those reporting they were currently taking ART but had missed taking pills in the previous seven days. Both outcome variables were binary.

We used 11 survey questions on HIV stigma to generate composite ‘yes/no’ binary variables for experienced community stigma, experienced health setting stigma and current internalised stigma. Composite variables were only generated for participants responding to all stigma questions contributing to that variable. Reponses on internalised stigma were given on a 4‐point Likert scale (0 = strongly disagree, 1 = disagree, 2 = agree and 3 = strongly agree) and later aggregated for each question (0/1 = disagree. 2/3 = agree). Questions on community and health setting stigma used pre‐coded response categories capturing the frequency of experiences during the last year (0 = never, 1 = once, 2 = a few times, 3 = often and 4 = not applicable because no one knows my status (‘never disclosed’)). Those responding ‘never’ or ‘never disclosed’ were categorised as ‘never experiencing either community or health setting stigma’. To create the three variables, respondents who disagreed or never experienced stigma on all the questions related to that variable were grouped as ‘never experiencing’ that type of stigma. Those agreeing or experiencing stigma on ≥1 question were categorised as ‘ever experiencing’ that type of stigma [18]. Our stigma measures were aligned with standardised measures that were approved by the UNAIDS’ monitoring and evaluation reference group (MERG) in 2014 [18, 28, 29].

*A priori* knowledge on risk factors for ART adherence informed decisions on other explanatory variables to explore for inclusion in analysis. We considered demographic variables (country, community/ study triplet, gender, age and marital status), socio‐economic factors (education, wealth, employment status and food security), mobility factors (nights spent away from home), behavioural factors (alcohol and drug use) and HIV‐specific factors (year of HIV diagnosis, time on ART, hiding pills (responding to the question ‘Have you ever hidden your ART pills so that others couldn’t see them’), HIV status disclosure and reason for starting ART). For alcohol use, we categorised respondents using scores from the WHO Alcohol Use Disorders Identification Test (AUDIT), [30] and for wealth, we used quintiles derived using principal component analysis. The group identified at lowest risk of the outcome was used as the reference category. Where this was unclear, we used the group with the largest numbers.

**Figure 1:**
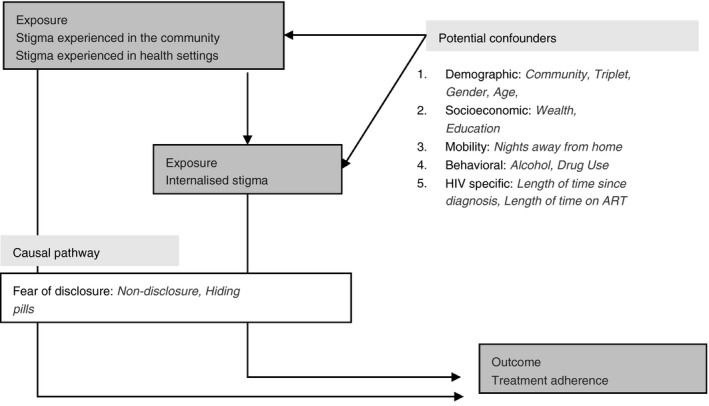
Conceptual framework.

We developed a conceptual framework (Figure 1) to structure our analysis using a hierarchical approach [31] based on previous work conceptualising HIV stigma [32] and associations between stigma and ART adherence [19]. We conducted analyses for the study population and then separately for each country.

We first described our study participants. Second, we described the distribution of ART adherence, HIV stigma and other explanatory variables. Third, we used logistic regression to estimate unadjusted associations between HIV stigma and ART adherence. We also estimated unadjusted associations between the other covariates and ART adherence and did the same for HIV stigma to understand potential confounding factors and identify variables to consider further in multivariable models. We conducted an analysis of the association between HIV stigma and ART adherence, stratified on the other explanatory variables that were considered *a priori* confounders and also those showing evidence of associations (*P* < 0.05) with adherence from our earlier unadjusted analysis.

Last, we conducted an adjusted analysis using multivariable logistic regression. We included groups of variables in our models in the stages identified in our conceptual framework, in order of their proximity to the outcome. Variables were included if they were considered potential confounders, either *a priori* and/or those showing an unadjusted association (*P* < 0.05) with the outcome. We excluded variables from our model if they were perceived to be on the causal pathway between stigma and ART adherence. To control for confounding by community‐level factors, we adjusted for study community (in Zambia) and study triplet (in South Africa) in all multivariable analysis. Study triplet was used instead of community in South Africa due to small numbers in the study population for several communities. The same series of models were built for each of the three stigma variables. We considered internalised stigma proximal to ART adherence and community and health setting stigma distal, adjusting a final set of models for each of the experienced stigmas (health setting and community) to account for this. We ran our models again with our restricted outcome definition (only those reporting they were currently taking ART but had missed taking pills in the previous seven days).

Written informed consent was obtained for all respondents enrolled in the population cohort. Ethics approval was obtained for the HPTN 071 (PopART) trial from the University of Zambia, Stellenbosch University, London School of Hygiene and Tropical Medicine.

## Results

**Figure 2:**
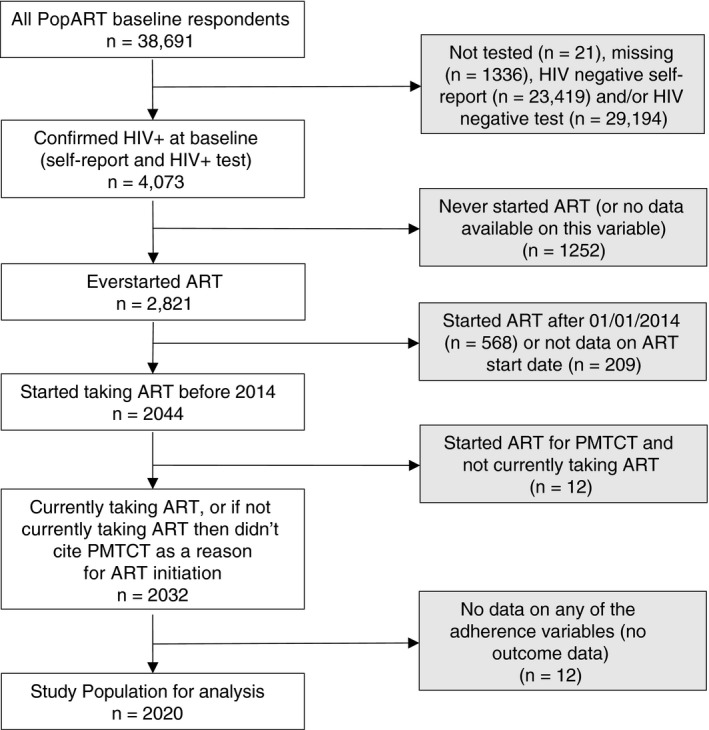
Study population

Our analysis initially included 2020 PLHIV (Zambia *n* = 1099; South Africa *n* = 921) (Figure 2). The number of individuals per community ranged from three to 250, with a higher proportion of women (88.6%) than men (11.4%). 76.6% of the study population were over the age of 30, and 6.3% aged 18–24 years. Approximately half the population (49%) were married or living as married, but with a higher proportion in Zambia (62.3%) than in South Africa (33.1%). Upper secondary school or University education was reached by 45.5% of respondents, although this proportion was notably higher in South Africa (70.1%) than Zambia (24.8%). Similar proportions of the study population were diagnosed with HIV each year, from before 2007 up until 2012. Only 6.4% of respondents were initiated on ART prior to 2005, with >60% starting ART after 2010 in both countries. Disclosure of HIV status (to friends, a religious leader, a health worker, family or a partner) was common, at 96.4% in Zambia and 97.7% in South Africa. 28% of the study population reported hiding their ART pills, with a higher proportion in Zambia (40.7%) than South Africa (12.9%). Missing data on all variables were minimal, ranging from 0 to 2.5% in Zambia and 0 to 2.7% in South Africa (Table 1).

**Table 1:** Study population characteristics

	Total study population			Zambia			South Africa	
	*n*	*n*/2020%		*n*		*n*/1099%	*n*	*n*/921%
Demographic characteristics								
Gender								
Female	1790		88.6%	950		86.4%	840	91.2%
Male	230		11.4%	149		13.6%	81	8.8%
Age								
18–24	128		6.3%	76		6.9%	52	5.6%
25–29	344		17.0%	165		15.0%	179	19.4%
30–34	521		25.8%	272		24.7%	249	27.0%
35–39	567		28.1%	310		28.2%	257	27.9%
>40	459		22.7%	275		25.0%	184	20.0%
Missing	1		0.0%	1		0.1%		0.0%
Study Triplet								
	–		–	258		23.5%	529	57.4%
	–		–	278		25.3%	292	31.7%
	–		–	291		26.5%	100	10.9%
	–		–	272		24.7%		
Marital status								
Married	990		49.0%	685		62.3%	305	33.1%
Divorced/Separated	246		12.2%	214		19.5%	32	3.5%
Widowed	146		7.2%	127		11.6%	19	2.1%
Never married	636		31.5%	73		6.6%	563	61.1%
Missing	2		0.1%	0		0.0%	2	0.2%
Socio‐economic characteristics								
Wealth quintile								
1 – Lowest	536		26.5%	295		26.8%	241	26.2%
2	426		21.1%	173		15.7%	253	27.5%
3	422		20.9%	219		19.9%	203	22.0%
4	408		20.2%	249		22.7%	159	17.3%
5 – Highest	223		11.0%	163		14.8%	60	6.5%
Missing	5		0.2%	0		0.0%	5	0.5%
Education								
None/Primary	558		27.6%	468		42.6%	90	9.8%
Lower Secondary	527		26.1%	354		32.2%	173	18.8%
Upper Secondary/University	919		45.5%	273		24.8%	646	70.1%
Missing	16		0.8%	4		0.4%	12	1.3%
Currently working								
No	1494		74.0%	802		73.0%	692	75.1%
Yes	526		26.0%	297		27.0%	229	24.9%
Food security								
No	1225		60.6%	605		55.1%	432	46.9%
Yes	793		39.3%	489		44.5%	487	52.9%
Missing	2		0.1%	5		0.5%	2	0.2%
Mobility characteristics								
Nights away from home†								
No	1685		83.4%	876		79.7%	809	87.8%
Yes	322		15.9%	216		19.7%	106	11.5%
Missing	13		0.6%	7		0.6%	6	0.7%
Behavioural characteristics								
Alcohol Audit score								
Score 0–7	1771		87.7%	967		88.0%	804	87.3%
Score 8–15	155		7.7%	85		7.7%	70	7.6%
Score 16+	42		2.1%	20		1.8%	22	2.4%
Missing	52		2.6%	27		2.5%	25	2.7%
Drug use (past 12 months)								
No	1988		98.4%	1076		97.9%	912	99.0%
Yes	22		1.1%	16		1.5%	6	0.7%
Missing	10		0.5%	7		0.6%	3	0.3%
HIV‐specific characteristics								
Year of HIV diagnosis								
Before 2009	421		20.8%	170	15.5%		251	27.3%
2007–2008	334		16.5%	176	16.0%		158	17.2%
2009–2010	438		21.7%	260	23.7%		178	19.3%
2011–2012	436		21.6%	261	23.8%		175	19.0%
2013–2014	275		13.6%	165	15.0%		110	11.9%
Missing	116		5.7%	67	6.1%		49	5.3%
First started ART								
1996–2005	130		6.4%	69	6.3%		61	6.6%
2006–2009	593		29.4%	323	29.4%		270	29.3%
2010–2011	500		24.8%	283	25.8%		217	23.6%
2012–2013	797		39.5%	424	38.6%		373	40.5%
Hiding pills								
No	1445		71.5%	645	58.7%		800	86.9%
Yes	566		28.0%	447	40.7%		119	12.9%
Missing	9		0.4%	7	0.6%		2	0.2%
HIV status disclosure								
Disclosed to anyone								
No	61		3.0%	40	3.6%		21	2.3%
Yes	1959		97.0%	1059	96.4%		900	97.7%
Disclosed to friends								
No	1711		84.7%	980	89.2%		731	79.4%
Yes	309		15.3%	119	10.8%		190	20.6%
Disclosed to religious leader								
No	1969		97.5%	1064	96.8%		905	98.3%
Yes	51		2.5%	35	3.2%		16	1.7%
Disclosed to health care worker								
No	1892		93.7%	1020	92.8%		872	94.7%
Yes	128		6.3%	79	7.2%		49	5.3%
Disclosed to family								
No	406		20.1%	235	21.4%		171	18.6%
Yes	1614		79.9%	864	78.6%		750	81.4%
Disclosed to partner								
No	1024		50.7%	505	46.0%		519	56.4%
Yes	996		49.3%	594	54.0%		402	43.6%
Primary reason for starting ART								
Started for PMTCT								
No	1760		87.1%	958	87.2%		802	87.1%
Yes	260		12.9%	141	12.8%		119	12.9%
Recommend by health worker								
No	1330		65.8%	616	56.1%		714	77.5%
Yes	690		34.2%	483	43.9%		207	22.5%
Started to protect partner								
No	1828		90.5%	973	88.5%		855	92.8%
Yes	192		9.5%	126	11.5%		66	7.2%
Started for own health								
No	938		46.4%	473	43.0%		465	50.5%
Yes	1082		53.6%	626	57.0%		456	49.5%

† >1 in the past 3 months.

Poor adherence to ART was reported by 320 (15.8%) respondents, with similar country‐specific findings (Zambia *n* = 186, 16.9%; SA *n* = 134, 14.5%). Most of those categorised as poor adherers reported *‘missing pills in the past seven days’* (*n* = 244). Thirty‐two respondents reported that they were not currently taking ART, and 80 respondents reported stopping in the previous 12 months. Poor adherence was slightly higher for men (18.7%) than women (15.5%), with similar distributions in each country (Table 2).

**Table 2:** Distribution of ART adherence and HIV stigma

ART Adherence		Total study population		Zambia		South Africa	
		*n*	*n*/2020%	*n*	*n*/1099%	*n*	*n*/921%
Currently taking ART	Yes	1988	98.4%	1092	99.4%	896	97.3%
	No	32	1.6%	7	0.6%	25	2.7%
Stopped ART in the past 12 months	Yes	80	4.0%	36	3.3%	44	4.8%
	No	1908	94.5%	1056	96.1%	852	92.5%
	Missing	32	1.6%	7	0.6%	25	2.7%
Missed pills in the past 7 days	Yes	244	12.1%	153	13.9%	91	9.9%
	No	1744	86.3%	939	85.4%	805	87.4%
	Missing	32	1.6%	7	0.6%	25	2.7%
ART adherence	Yes	1700	84.2%	913	83.1%	787	85.5%
	No	320	15.8%	186	16.9%	134	14.5%
HIV Stigma							
I have lost respect or standing in the community because of my HIV status	Disagree	1732	85.7%	919	83.6%	813	88.3%
	Agree	258	12.8%	161	14.6%	97	10.5%
	Missing	30	1.5%	19	1.7%	11	1.2%
I think less of myself	Disagree	1763	87.3%	952	86.6%	811	88.1%
	Agree	240	11.9%	137	12.5%	103	11.2%
	Missing	17	0.8%	10	0.9%	7	0.8%
I have felt ashamed because of my HIV status	Disagree	1758	87.0%	945	86.0%	813	88.3%
	Agree	242	12.0%	141	12.8%	101	11.0%
	Missing	20	1.0%	13	1.2%	7	0.8%
Internalised Stigma	No	1552	76.8%	819	74.5%	733	79.6%
	Yes	434	21.5%	257	23.4%	177	19.2%
	Missing	34	1.7%	23	2.1%	11	1.2%
People have talked badly about me because of my HIV status	None	1617	80.0%	846	77.0%	771	83.7%
	Some	382	18.9%	238	21.7%	144	15.6%
	Missing	21	1.0%	15	1.4%	6	0.7%
I have been verbally insulted, harassed and/or threatened because of my HIV status	None	1803	89.3%	972	88.4%	831	90.2%
	Some	200	9.9%	116	10.6%	84	9.1%
	Missing	17	0.8%	11	1.0%	6	0.7%
I have been physically assaulted because of my HIV status	None	1899	94.0%	1046	95.2%	853	92.6%
	Some	106	5.2%	43	3.9%	63	6.8%
	Missing	15	0.7%	10	0.9%	5	0.5%
Someone else disclosed my HIV status without my permission	None	1682	83.3%	904	82.3%	778	84.5%
	Some	314	15.5%	184	16.7%	130	14.1%
	Missing	24	1.2%	11	1.0%	13	1.4%
I have felt that people have not wanted to sit next to me because of my HIV status	None	1915	94.8%	1060	96.5%	855	92.8%
	Some	89	4.4%	31	2.8%	58	6.3%
	Missing	16	0.8%	8	0.7%	8	0.9%
Experienced stigma in the community	No	1468	72.7%	764	69.5%	704	76.4%
	Yes	519	25.7%	317	28.8%	202	21.9%
	Missing	33	1.6%	18	1.6%	15	1.6%
Healthcare workers talked badly about me because of my HIV status	Disagree	1905	94.3%	1050	95.5%	855	92.8%
	Agree	99	4.9%	39	3.5%	60	6.5%
	Missing	16	0.8%	10	0.9%	6	0.7%
A health worker disclosed my HIV status without my permission	Disagree	1909	94.5%	1054	95.9%	855	92.8%
	Agree	91	4.5%	35	3.2%	56	6.1%
	Missing	20	1.0%	10	0.9%	10	1.1%
I have been denied health services because of my HIV status	Disagree	1939	96.0%	1081	98.4%	858	93.2%
	Agree	65	3.2%	10	0.9%	55	6.0%
	Missing	16	0.8%	8	0.7%	8	0.9%
Experienced stigma in health settings	Disagree	1844	91.3%	1020	92.8%	824	89.5%
	Agree	152	7.5%	66	6.0%	86	9.3%
	Missing	24	1.2%	13	1.2%	11	1.2%

Stigma experienced in the community was most frequently reported (overall 25.7%; Zambia 28.8%; SA 21.9%), then internalised stigma (overall 21.5%; Zambia 23.4%; SA 19.2%). Stigma experienced in health care settings was less frequently reported (overall 7.5%; Zambia 6%; SA 9.3%) (Table 2).

Among the total study population, those reporting stigma experienced in the community or internalised stigma were more likely to be non‐adherent than those who did not, with unadjusted ORs of 1.68 (95% CI 1.29–2.18, *P* < 0.001) and 1.52 (95% CI 1.15–2.01, *P* = 0.003), respectively. Those experiencing health setting stigma were only slightly more likely to be non‐adherent to ART than those who did not (OR 1.19, 95% CI 0.76–1.85, *P* = 0.45). Country‐specific estimates were similar. In Zambia, those experiencing community stigma had 1.89 (95% CI 1.35–2.65, *P* < 0.001) the odds of poor adherence, and those reporting internalised stigma 1.62 (95% CI 1.13–2.3, *P* = 0.008) the odds of poor adherence. In South Africa, the association between each of community and internalised stigma and poor adherence gave ORs of 1.32 (95% CI 0.85–2.05, *P* = 0.22) and 1.34 (95% CI 0.85–2.11, *P* = 0.21), respectively (Table 4).

In the total study population, poor ART adherence was associated with explanatory variables including community/triplet (*P* < 0.001), higher alcohol consumption (*P* < 0.001), lower educational attainment (*P* = 0.04), increased mobility (*P* < 0.001) and hiding pills (*P* = 0.03). Of these, community/triplet showed strong evidence of an association with all three stigma variables (all *P* < 0.001). Higher alcohol consumption was associated with internalised stigma (*P* < 0.001), and hiding pills was associated with both internalised and health setting stigma (*P* < 0.001 and *P* = 0.02, respectively), but there was no evidence of an association with experienced community stigma (*P* = 0.73). These associations differed slightly in each country, for example, there was evidence that education was associated with poor adherence in South Africa but not Zambia and mobility in Zambia but not South Africa (Table 3).

**Table 3:** Univariable logistic regression estimates of odds ratios for each variable with ART adherence

	Study Population (*N* = 2020)	Non‐adherence (*n* = 320)	%	OR	95% CI	*P*‐value†
Demographic						
Gender						
Female	1790	277	15.5%	1		0.22
Male	230	43	18.7%	1.26	(0.88–1.79)	
Age						
18–24	128	19	14.8%	0.97	(0.56–1.68)	0.50
25–29	344	66	19.2%	1.32	(0.91–1.91)	
30–34	521	79	15.2%	0.99	(0.70–1.41)	
35–39	567	86	15.2%	0.99	(0.71–1.40)	
>40	459	70	15.3%	1		
Study Triplet						
Zambia – 1	258	53	20.5%	1		<0.001
Zambia – 2	278	46	16.5%	0.77	(0.50–1.19)	
Zambia – 3	291	63	21.6%	1.07	(0.71–1.61)	
Zambia – 4	272	24	8.8%	0.37	(0.22–0.63)	
SA – 5	529	65	12.3%	0.54	(0.36–0.81)	
SA – 6	292	49	16.8%	0.78	(0.51–1.20)	
SA – 7	100	20	20.0%	0.97	(0.54–1.72)	
Socio‐economic						
Wealth quintile						
1 – Lowest	536	83	15.5%	1		0.06
2	426	70	16.4%	1.07	(0.76–1.52)	
3	422	50	11.8%	0.73	(0.50–1.07)	
4	408	73	17.9%	1.19	(0.84–1.68)	
5 – Highest	223	44	19.7%	1.34	(0.90–2.01)	
Missing	5	5				
Education						
None/Primary	558	84	15.1%	1		0.04
Lower Secondary	527	103	19.5%	1.37	(1.00–1.88)	
Upper Secondary/University	919	133	14.5%	0.95	(0.71–1.28)	
Mobility						
Nights away						
No	1685	249	14.8%	1		0.002
Yes	322	71	22.0%	1.63	(1.21–2.19)	
Behavioural						
Alcohol Audit score‡						
Score 0–7	1771	253	14.3%	1		<0.001
Score 8–15	155	40	25.8%	2.09	(1.42–3.06)	
Score 16+	42	14	33.3%	3.00	(1.56–5.78)	
Drug use (past 12 months)						
No	1988	308	15.5%	1		0.06
Yes	22	7	31.8%	2.55	(1.03–6.29)	
HIV‐specific						
Hiding pills						
No	1445	212	14.7%	1		0.03
Yes	566	105	18.6%	1.32	(1.02–1.71)	
HIV status disclosure						
No	61	12	19.7%	1		0.42
Yes	1959	308	15.7%	0.76	(0.40–1.45)	
Year of HIV diagnosis						
Before 2007	421	64	15.2%	1		0.43
2007–2008	334	45	13.5%	0.87	(0.58–1.31)	
2009–2010	438	70	16.0%	1.06	(0.73–1.53)	
2011–2012	436	80	18.3%	1.25	(0.87–1.80)	
2013–2014	275	47	17.1%	1.15	(0.76–1.74)	
First started ART						
1996–2005	130	20	15.4%	0.87	(0.52–1.45)	0.46
2006–2009	593	84	14.2%	0.79	(0.59–1.06)	
2010–2011	500	78	15.6%	0.88	(0.65–1.20)	
2012–2013	797	138	17.3%	1		

† LRT for the overall association of the variable with ART adherence.

‡ Low dependence 0–7, medium dependence 8–15, high dependence 16+.

Stigma experienced in the community was more likely to be reported by those who had disclosed their HIV status to their family (OR 1.42 95% CI 1.08–1.87, *P* = 0.01) or friends (OR 1.38 95% CI 1.05–1.81, *P* = 0.02). There was little evidence that food security was associated with ART adherence (OR 1.03 95% CI 0.75–1.42, *P* = 0.83), but strong evidence that those experiencing HIV stigma were more likely to be food insecure than those who did not (community, OR 1.88, 95% CI 1.53–2.32, *P* < 0.001, internalised, OR 1.72 95% CI 1.38–2.14, *P* < 0.001 and health setting, OR 95% CI, *P* = 0.02).

Multivariable analysis was restricted to individuals with complete data on all variables (Total *n* = 1888; Zambia *n* = 1034, South Africa *n* = 854). After adjusting for the potential confounding effects of demographic, socio‐economic, mobility and behavioural factors and for the other domains of stigma in line with our conceptual framework, there remained strong evidence of an association between experienced community stigma and ART adherence (aOR 1.63, 95% CI 1.21−2.19, *P* = 0.001) but not internalised stigma and ART adherence (aOR 1.31, 95% CI 0.96–1.79, *P* = 0.09) or health setting stigma and ART adherence (aOR 1.05; 95% CI 0.64–1.72; *P* = 0.86) (Table 4).

**Table 4:** Univariable and multivariable logistic regression estimates of odds ratios for each stigma variable and ART adherence

	ART adherence		Unadjusted models			Adjusted models§			Adjusted models¶		
	*n*/*N* †	%	OR	95% CI	P_w_	aOR	95% CI	P_w_	aOR	95% CI	P_w_
Total Study Population	*N* = 2020		Analysis restricted to *n* = 1888‡								
Experienced stigma in the community											
No	201/1468	13.7%	1			1			1		
Yes	110/519	21.2%	1.68	(1.29–2.19)	<0.001	1.65	(1.25–2.18)	<0.001	1.63	(1.21–2.19)	0.001
Experienced stigma in health settings											
No	290/1844	15.7%	1			1			1		
Yes	27/152	17.8%	1.19	(0.76–1.86)	0.44	1.38	(0.87–2.20)	0.17	1.05	(0.64–1.72)	0.86
Internalised Stigma											
No	228/1552	14.7%	1			1			1		
Yes	87/434	20.0%	1.51	(1.15–2.00)	0.004	1.50	(1.12–2.01)	0.007	1.31	(0.96–1.79)	0.09
Zambia	*N* = 1099		Analysis restricted to *n* = 1034‡								
Experienced stigma in the community											
No	106/764	13.9%	1			1			1		
Yes	75/317	23.7%	1.89	(1.35–2.65)	<0.001	1.98	(1.38–2.83)	<0.001	2.03	(1.40–2.94)	<0.001
Experienced stigma in health settings											
No	174/1020	17.1%	1			1			1		
Yes	11/66	16.7%	0.99	(0.51–1.94)	0.98	1.10	(0.55–2.22)	0.79	0.80	(0.39–1.65)	0.54
Internalised Stigma											
No	125/819	15.3%	1			1			1		
Yes	58/257	22.6%	1.62	(1.13–2.31)	0.008	1.67	(1.15–2.44)	0.007	1.44	(0.97–2.14)	0.07
South Africa	*N* = 921		Analysis restricted to *n* = 854‡								
Experienced stigma in the community											
No	95/704	13.5%	1			1			1		
Yes	35/202	17.3%	1.32	(0.85–2.05)	0.22	1.21	(0.76–1.93)	0.43	1.01	(0.58–1.74)	0.98
Experienced stigma in health settings											
No	116/824	14.1%	1			1			1		
Yes	16/86	18.6%	1.45	(0.80–2.64)	0.22	1.67	(0.89–3.13)	0.11	1.66	(0.79–3.47)	0.18
Internalised Stigma											
No	103/733	14.1%	1			1			1		
Yes	29/177	16.4%	1.34	(0.85–2.11)	0.21	1.41	(0.87–2.27)	0.16	1.31	(0.78–2.21)	0.30

† *n* = non‐adherent; *N* = total individuals reporting ever starting ART.

‡ Analysis restricted to respondents with complete data on community/triplet, gender, age, education, wealth, mobility, alcohol and all stigma variables.

§ Adjusted for community/triplet, gender, age, education, wealth, mobility, alcohol.

¶ Adjusted for community/triplet, gender, age, education, wealth, mobility, alcohol and experienced stigma (internalised stigma adjusted for community and health setting stigma; health setting stigma adjusted for community stigma; community stigma adjusted for health setting stigma.

In Zambia, there was strong evidence of an association between stigma experienced in the community poor adherence (aOR 2.03, 95% CI 1.40–2.94, *P* < 0.001), weak evidence of an association between internalised stigma and poor adherence (aOR 1.44; 95% CI 0.97–2.14; *P* = 0.09) and no evidence of an association between health setting stigma and poor adherence (aOR 0.80; 95% CI 0.39–1.65; *P* = 0.54) (Table 4).

In South Africa, there was a stronger association between health setting stigma and ART adherence than in Zambia, although the evidence for this association was weak (aOR 1.66 95% CI 079–3.47, *P* = 0.18). For community and internalised stigma, odds ratios were close to 1, and there was no evidence of associations with either (Table 4).

Although the odds of poor adherence for those reporting stigma experienced in the community were different in each country (aOR 2.03 in Zambia vs aOR 1.01 in South Africa), there was only weak evidence that these associations were different (*P* = 0.08). There was no evidence that the associations for health setting stigma and ART adherence (*P* = 0.38) and internalised stigma and ART adherence (*P* = 0.57) differed in Zambia and South Africa.

We conducted further analysis, restricting our outcome to individuals reporting they were currently on ART (*n* = 1861) and defining non‐adherence as missing pills in the previous 7 days. Findings from our adjusted models for the whole study population were similar to our primary definition of ART adherence (community stigma aOR 1.60 95% CI 1.15–2.22 *P* = 0.005, internalised stigma aOR 1.28 95% CI 0.90–1.81, *P* = 0.17; health setting stigma aOR 0.86 96% CI 0.48–1.53 *P* = 0.60) (Table S1).

## Discussion

Among a large population sample of PLHIV reporting ever taking ART in the 21 communities included in the HPTN 071 (PopART) study in Zambia and South Africa, 16% reported one or more of missing pills in the previous seven days (12%), currently taking ART but having stopped during the previous 12 months (4%), or no longer taking ART (2%). Approximately 25% reported ever experiencing community stigma, 20% internalised stigma and 8% health setting stigma. PLHIV reporting stigma experienced in the community were more than 1.5 times more likely to report poor ART adherence than those who did not.

In Zambia, participants reporting experiences of community stigma were twice as likely to report poor adherence as those who did not, but we saw no such association in South Africa. Although there was only weak evidence that these associations were different in each country, it is also possible that they represent the different contexts. HIV stigma and poor adherence were both more common in Zambian than South African study communities. In the South Africa, a strong history of community led HIV treatment advocacy and awareness could have mitigated HIV stigma and its effect on ART adherence.

Health setting stigma was less frequently reported and may play a less important role in adherence because people generally take their pills away from a health facility. In both countries, the association between internalised stigma and ART adherence was partly explained after adjustments were made for experienced stigma in community or health settings. We hypothesised that stigma experienced in the community may itself cause internalised stigma.

Our findings are similar to previous cross‐sectional studies looking at stigma and ART adherence [19, 20, 21, 22, 23, 24, 25], yet direct comparisons are challenging due to variation in the specific measures used to look at these concepts. Variation also exists in the statistical adjustments made when investigating these associations. We made our own theoretical assumptions on factors to include in our multivariable models. Alcohol was considered a potential confounder, as it has been in other studies exploring these associations [19, 22, 33]. Some studies have, however, identified alcohol as a means of coping with HIV status [19], compromising ability to adhere to treatment. Similarly, wealth was treated as a confounding factor in our analysis, but the relationship between economic security and HIV‐related stigma is likely to be more complicated and potentially *‘mutually reinforcing’* [19]. We did not treat hiding pills and HIV status disclosure as confounders in our multivariable models as we suggest these variables lie on the causal pathway between experience of stigma and ART adherence. Including either of these variables in our models made little difference to the associations we saw between stigma and ART adherence. Hiding pills has been frequently reported in Zambia and South Africa [34] and, with strong unadjusted associations seen in this study, would be useful to explore in further work on stigma related to HIV treatment.

Ours was a large study, and we used validated measures of HIV stigma [29] and measured a large number of characteristics providing the opportunity for a thorough assessment of potential confounding. We looked at the association between three stigma ‘domains’ on adherence to ART, giving an opportunity to identify the specific areas of stigma that had the strongest associations with ART adherence. We interpreted our findings based on a conceptual framework that considered some of the latest thinking on HIV stigma, enabling wider comparison and contributing to existing work in this field. A composite measure of ART adherence was used to ensure inclusion of poor adherence over a year, in line with our stigma measures. In a systematic review of self‐report measures, seven‐day recall was most commonly used and considered effective due to the inclusion of a shorter time period, whilst covering a weekend (where adherence is often lower), but longer recall also considered important for allowing greater variability in adherence [35]. We acknowledge that our composite adherence outcome could measure slightly different concepts, but tested this using a restricted outcome in our analysis and found similar results. There were relatively few missing data.

There were also limitations. Our study communities were purposively sampled, and although we consider our findings generalisable to socio‐economically disadvantaged, peri‐urban communities with high HIV prevalence in Zambia and the Western Cape of South Africa [27, 36], the generalisability of our findings to other sub‐Saharan African settings may be limited. The greater proportion of women in our study population was reflective of the overall population cohort and the higher HIV prevalence among women (26%) than men (12%) [27], rather than a selection bias among individuals who had ever taken ART. Yet, this disparity limits the generalisability of our findings to men, who in previous research have shown worse ART adherence than women [15, 37]. Our analysis excluded individuals who were not aware of or not willing to report their HIV status and those who reported no date for starting ART. Experiences of stigma may have been different among those not willing to disclose their HIV status to our research team and may have led to an underestimation of HIV stigma and of its association with ART adherence. Underreporting of poor ART adherence was possible due to it being contrary to clinical guidance. However, the extent of underreporting to our research team was unlikely to differ according to an individual's experience of stigma, and so, it is unlikely to have introduced bias to our findings. Our findings of approximately 84% adherence are compatible with viral suppression data on a random subsample of individuals who were HIV‐positive at the time of the baseline survey; these data indicated that approximately 90% of HIV‐positive individuals who were taking ART were virally suppressed [27]. Other factors also relied on self‐report and were potentially prone to either under or over‐reporting (e.g. alcohol consumption and wealth). Stigma questions specifically relating to HIV treatment [38] may have given a more specific indication of mechanisms for non‐adherence and would be useful for consideration in future work.

## Conclusions

Our analysis has provided additional evidence that HIV‐related stigma is associated with poor ART adherence and has identified the relative importance of the different types and components of stigma among a large sample of PLHIV across 21 communities in Zambia and South Africa. If we are to reach viral suppression among 90% of people on ART by 2020 and 95% by 2030, it will be important to learn whether interventions that reduce HIV stigma could also improve lifelong adherence to ART.

## Supporting information


**Table S1.** Univariable and multivariable logistic regression estimates of odds ratios for each stigma variable and missing ART pills in the previous 7 days.
