# Supplementary material for: Association between HIV stigma and antiretroviral therapy adherence among adults living with HIV: baseline findings from the HPTN 071 (PopART) trial in Zambia and South Africa
Source: Trop Med Int Health. 2020 Aug 26;25(10):1246–60. doi: 10.1111/tmi.13473 (PMC7590062; doi:10.1111/tmi.13473)
Supplement: Supplementary file 1 — Table S1. Univariable and multivariable logistic regression estimates of odds ratios for each stigma variable and missing ART pills in the previous 7 days. [file TMI-25-1246-s001.docx]

| **Supplementary Table 1:** Univariable and multivariable logistic regression estimates of odds ratios for each stigma variable and missing ART pills in the previous 7 days | | | | | | | | | | | |
| --- | --- | --- | --- | --- | --- | --- | --- | --- | --- | --- | --- |
|  | ART adherence | | Unadjusted models | | | Adjusted models^§^ | | | Adjusted models^¶^ | | |
|  | n/N ^†^ | % | OR | 95% CI | P*_w_* | aOR | 95% CI | P*_w_* | aOR | 95% CI | P*_w_* |
| **Total Study Population** | N=1956 |  | analysis restricted to n=1861^‡^ | | | | | | | | |
| Experienced stigma in the community | |  |  |  |  |  |  |  |  |  |  |
| No | 153/1281 | 10.60% | 1 |  |  | 1 |  |  | 1 |  |  |
| Yes | 83/429 | 16.21% | 1.61 | (1.20-2.17) | 0.001 | 1.55 | (1.14-2.12) | 0.006 | 1.6 | (1.15-2.22) | 0.005 |
| Experienced stigma in health settings | |  |  |  |  |  |  |  |  |  |  |
| No | 224/1594 | 12.32% | 1 |  |  | 1 |  |  | 1 |  |  |
| Yes | 18/129 | 12.24% | 0.99 | (0.59-1.68) | 0.98 | 1.12 | (0.64-1.93) | 0.69 | 0.86 | (0.48-1.53) | 0.6 |
| Internalised Stigma |  |  |  |  |  |  |  |  |  |  |  |
| No | 176/1351 | 11.53% | 1 |  |  | 1 |  |  | 1 |  |  |
| Yes | 65/364 | 15.15% | 1.41 | (1.03-1.94) | 0.03 | 1.42 | (1.02-1.97) | 0.04 | 1.28 | (0.90-1.81) | 0.17 |
| **Zambia** | N=1074 |  | analysis restricted to n=1029^‡^ | | | | | | | | |
| Experienced stigma in the community | |  |  |  |  |  |  |  |  |  |  |
| No | 87/672 | 11.46% | 1 |  |  | 1 |  |  | 1 |  |  |
| Yes | 61/254 | 19.37% | 1.82 | (1.27-2.62) | 0.001 | 1.8 | (1.22-2.64) | 0.003 | 1.89 | (1.27-2.80) | 0.002 |
| Experienced stigma in health settings | |  |  |  |  |  |  |  |  |  |  |
| No | 144/870 | 14.20% | 1 |  |  | 1 |  |  | 1 |  |  |
| Yes | 9/57 | 12.31% | 0.86 | (0.40-1.85) | 0.71 | 0.89 | (0.40-1.96) | 0.76 | 0.66 | (0.29-1.50) | 0.32 |
| Internalised Stigma |  |  |  |  |  |  |  |  |  |  |  |
| No | 104/711 | 12.76% | 1 |  |  | 1 |  |  | 1 |  |  |
| Yes | 47/208 | 18.43% | 1.53 | (1.04-2.25) | 0.03 | 1.59 | (1.06-2.40) | 0.03 | 1.42 | (0.92-2.18) | 0.11 |
| **South Africa** | N=882 |  | analysis restricted to n=832^‡^ | | | | | | | | |
| Experienced stigma in the community | |  |  |  |  |  |  |  |  |  |  |
| No | 66/619 | 9.64% | 1 |  |  | 1 |  |  | 1 |  |  |
| Yes | 22/175 | 11.17% | 1.15 | (0.68-1.96) | 0.6 | 1.13 | (0.65-2.00) | 0.66 | 1.01 | (0.52-1.95) | 0.97 |
| Experienced stigma in health settings | |  |  |  |  |  |  |  |  |  |  |
| No | 80/724 | 9.95% | 1 |  |  | 1 |  |  | 1 |  |  |
| Yes | 10/72 | 12.20% | 1.26 | (0.60-2.62) | 0.54 | 1.39 | (0.65-3.01) | 0.4 | 1.38 | (0.56-3.41) | 0.48 |
| Internalised Stigma |  |  |  |  |  |  |  |  |  |  |  |
| No | 72/640 | 10.11% | 1 |  |  | 1 |  |  | 1 |  |  |
| Yes | 18/156 | 10.34% | 1.14 | (0.65-1.99) | 0.63 | 1.24 | (0.70-2.21) | 0.46 | 1.19 | (0.64-2.21) | 0.59 |
| ^†^  n=missed ART pills in the past 7 days; N=total individuals reporting ever starting ART | | | | | | | | | | | |
| ^‡^ analysis restricted to respondents with complete data on community/triplet, gender, age, education, wealth, mobility, alcohol and all stigma variables | | | | | | | | | | | |
| ^§^ adjusted for community/triplet, gender, age, education, wealth, mobility, alcohol | | | | | | | | | | | |
| ^¶^ adjusted for community/triplet, gender, age, education, wealth, mobility, alcohol and experienced stigma (internalised stigma adjusted for community and health setting stigma; health setting stigma adjusted for community stigma; community stigma adjusted for health setting stigma | | | | | | | | | | | |
